# Neural differentiation of transplanted neural stem cells in a rat model of striatal lacunar infarction: light and electron microscopic observations

**DOI:** 10.3389/fncel.2012.00030

**Published:** 2012-08-02

**Authors:** Vilma C. Muñetón-Gómez, Ernesto Doncel-Pérez, Ana P. Fernandez, Julia Serrano, Andrea Pozo-Rodrigálvarez, Lara Vellosillo-Huerta, Julian S. Taylor, Gloria P. Cardona-Gómez, Manuel Nieto-Sampedro, Ricardo Martínez-Murillo

**Affiliations:** ^1^Neurovascular Research Group, Department of Molecular, Cellular, and Developmental Neurobiology, Spanish Council for Scientific Research (CSIC), Instituto CajalMadrid, Spain; ^2^Grupo de Química Neuro-regenerativa, Unidad de Neurología Experimental, Hospital Nacional de Parapléjicos de Toledo (SESCAM)Toledo, Spain; ^3^Grupo de Neurociencias, Sede de Investigación Universitaria, Facultad de Medicina, Universidad de AntioquiaMedellín, Colombia; ^4^Grupo de Plasticidad Neural, Department of Functional and Systems Neurobiology, Spanish Council for Scientific Research (CSIC), Instituto CajalMadrid, Spain

**Keywords:** electron microscopy, immunohistochemistry, neural grafting, neural stem cells, striatum, stroke, dopaminergic, vascular parkinsonism

## Abstract

The increased risk and prevalence of lacunar stroke and Parkinson's disease (PD) makes the search for better experimental models an important requirement for translational research. In this study we assess ischemic damage of the nigrostriatal pathway in a model of lacunar stroke evoked by damaging the perforating arteries in the territory of the substantia nigra (SN) of the rat after stereotaxic administration of endothelin-1 (ET-1), a potent vasoconstrictor peptide. We hypothesized that transplantation of neural stem cells (NSCs) with the capacity of differentiating into diverse cell types such as neurons and glia, but with limited proliferation potential, would constitute an alternative and/or adjuvant therapy for lacunar stroke. These cells showed neuritogenic activity *in vitro* and a high potential for neural differentiation. Light and electron microscopy immunocytochemistry was used to characterize GFP-positive neurons derived from the transplants. 48 h after ET-1 injection, we characterized an area of selective degeneration of dopaminergic neurons within the nigrostriatal pathway characterized with tissue necrosis and glial scar formation, with subsequent behavioral signs of Parkinsonism. Light microscopy showed that grafted cells within the striatal infarction zone differentiated with a high yield into mature glial cells (GFAP-positive) and neuron types present in the normal striatum. Electron microscopy revealed that NSCs-derived neurons integrated into the host circuitry establishing synaptic contacts, mostly of the asymmetric type. Astrocytes were closely associated with normal small-sized blood vessels in the area of infarct, suggesting a possible role in the regulation of the blood brain barrier and angiogenesis. Our results encourage the use of NSCs as a cell-replacement therapy for the treatment of human vascular Parkinsonism.

## Introduction

Stroke is the second most common cause of death and major cause of disability worldwide, and most therapies have focused on early neuroprotection as a treatment strategy (Sahota and Savitz, 2011). Lacunar infarcts caused by occlusion of deep penetrating branches of major cerebral arteries are responsible for about 25% of all strokes (Sudlow and Warlow, 1997) and may cause idiopathic Parkinson's disease (PD), particularly in the elderly and as a consequence of drug abuse (Winslow et al., 2007). Between 4.4–12% of all cases of Parkinsonism can be accounted by stroke (Baldereschi et al., 2000). The increased risk and prevalence of lacunar stroke with PD (Becker et al., 2010) makes the search for experimental translational models necessary (Bailey et al., 2009; Martinez-Murillo et al., 2009). In this paper ischemic damage of the nigrostriatal path as a model of lacunar stroke was performed by affecting perforating arteries in the rat substantia nigra (SN) territory, following stereotaxic administration of the potent vasoconstrictor peptide endothelin-1 (ET-1) in the rat (Sharkey et al., 1993). Mechanisms of sensorimotor neurological deficit after ischemic injury include an increased sensitivity of cerebral arteries to vasoconstriction and vasospasm (Hansen-Schwartz et al., 2007). Peptide ET-1 mediates a strong contractile effect on cerebral arteries, through the ETa subtype receptor which are mainly located on smooth muscle cells, whereas a vasodilatory response is mediated by ETb receptor activation located in endothelial cells (Nilsson et al., 1997; Szok et al., 2001). The ET system is widely expressed in the brain (Schinelli, 2006), including a few dopaminergic (DA) neurons in the SN pars reticulata (Sluck et al., 1999). However, the effect of pharmacological modulation of the ET system on brain function or pathophysiology is still poorly understood (Schinelli, 2006). Many pathological conditions of the human brain, including infarcts and lacunae, have been related to altered expression of the ET system (Nie and Olsson, 1996). Experimentally local administration of ET-1 in the brain produces a dramatic decrease in blood flow leading to focal ischemic insult (Dreier et al., 2007). In the striatum, the area of ischemia can be detected using *in vivo* Magnetic Resonance Imaging (MRI) (Martinez-Murillo et al., 2007, 2009).

In this study ET-1 was injected into the SN to induce ischemic cerebral vasoconstriction, an original experimental animal model for inducing *lacunae* ictus in stroke research. This model facilitates characterization of the area of selective DA neuronal degeneration within the nigrostriatal pathway, including tissue necrosis, inflammation, glial scar formation, and behavioral signs of Parkinsonism. Previous experimental studies of cellular transplantation with embryonic neural stem cells (ESCs) have demonstrated their potential to differentiate into several cell types within the central nervous system and replacement of nerve cells destroyed in the striatum after a vascular compromise (Takagi et al., 2005; Buhnemann et al., 2006) or Parkinsonian diseases (Studer et al., 1998; Kim et al., 2002). The therapeutic potential of transplantation of NSCs from transgenic embryos expressing green fluorescent protein (GFP) is assessed for lacunar stroke in this study. A previous study has characterized these cells by microarray analysis and *in vitro* neuritogenic activity as demonstrating a high potential for neural differentiation (Doncel-Pérez et al., 2009). We show that transplanted NSCs survived within the infarct area, and differentiated into neuronal and glial cells, specifically into striatal non-DA neurons and astrocytes. NSCs-derived neurons integrated into the host circuitry by establishing synaptic contacts, while GFP-positive astrocytes were detected lying directly onto small-sized blood vessel within the infarct area. Finally NSCs transplantation promoted recovery of voluntary limb as shown by using standard protocols described in other rat models of cerebral infarction (Bederson et al., 1986) and PD (Lundblad et al., 2002).

## Materials and methods

### Animals

Male Wistar rats (*n* = 32) provided by Harlan (Barcelona, Spain) weighing 250–350 g were housed under controlled light, temperature, and relative humidity. The animals had free access to food and water. All procedures were carried out in accordance with the European Communities Council Directive (86/609/EEC) on animal experiments, taking special care to minimize pain and discomfort to the experimental animals, under a protocol approved by the Animal Welfare Committee of the Instituto Cajal (CSIC) which adheres to the recommendations of the European Council and Spanish Department of Health for Laboratory Animals (Real Decreto 1201/2005).

### Ischemia model

Experiments were performed in each of the following four groups: (a) non-injured, six animals; (b) sham operated with intracranial injection of phosphate buffered saline (PBS), six animals; (c) intranigral ET-1 injection, ten animals; and (d) intranigral ET-1 injection with NSCs transplantation, 10 animals. Rats were anesthetized with 2-chloro-2-(difluoromethoxy)-1,1,1-trifluoro-ethane (Isoflurane, Baxter S. L., Valencia, Spain) with a vaporizer from MSS International Ltd. (Keighley, UK) and secured to a stereotaxic instrument (David Kopf Instruments, Tujunga, CA). Deep anesthesia was confirmed and monitored by the absence of nociceptive pain reflexes. Intra-cerebral injections of either vehicle or ET-1 were performed using a 10 μl Hamilton syringe with a 34 gauge blunt needle. The dura was removed and 0.05 μg of ET-1 (Calbiochem, La Jolla, CA) diluted in 1 μl of PBS were injected at a rate of 1 μl/3 min within the SN, specifically at the coordinates AP, −5.8; L, −2, and V, −8.4 (Paxinos et al., 1980).

### Determination of infarct size

Studies were performed for three animals of each experimental group, two days after ET-1 intranigral injection. Infarct size was determined in different types of lesions with *in vivo* MRI (see below) (Martinez-Murillo et al., 2007) or following post-mortem staining with 2,3,5-triphenyltetrazolium chloride (TTC) (Bederson et al., 1986). The TTC staining was performed in animals following euthanasia with an overdose of sodium pentobarbital. Brains were rapidly removed and cut at 4°C into serial 1 mm-thick coronal slices (5–10 from the frontal tips) using stainless matrices (Brain Matrix, WPI, UK) and then stained with TTC [1% in 0.1 M phosphate buffer (PB)]. Infarct volumes were calculated by sampling each side of the coronal sections with a digital camera (Nikon Coolpix 990) to produce images of the fresh brain slices. With the aid of an image analysis system (AIS, Imaging Research Inc., Linton, England), as described earlier (Granado et al., 2008), two independent observers empirically determined a density threshold for gray-scale level staining, and the images were analyzed using the ImageJ 1.33 u program (National Institutes of Health, Bethesda, MD). The contralateral hemisphere perimeter was overlapped onto the ipsilateral hemisphere to exclude edema, and infarct borders were delineated with an operator controlled cursor. The area of infarct, which was unstained, was determined by counting the pixels contained within the outlined regions of interest and expressed in square millimeters. Infarct volumes (in mm^3^) were integrated from the infarct areas over the extent of the infarct calculated as an orthogonal projection. Statistical significance level was obtained by One-Way ANOVA using the SigmaStat (version 2.0) software. *P*-values less than 0.05 were considered statistically significant.

### Magnetic resonance imaging (MRI)

Infarct volume was analyzed by MRI at 48 h after surgery, following a procedure previously described by our group (Martinez-Murillo et al., 2007). Images were acquired with a Bruker Biospec 47/40 spectrometer, with a main field of 4.7 T and a 40 cm bore. This magnet is equipped with an unshielded gradient coil with a maximum strength of 372 mT/m. The RF coil, acting as a transmitter and receiver, was a 30 mm-diameter surface coil placed just on top of the head of the rat. Initially, localization fast spin echo images were acquired both in the coronal and in the sagittal directions, and coronal slices were selected for imaging. Diffusion-weighted images were acquired with a 30 × 30 mm field of view, a slice thickness of 1 mm, and a 256 × 64 acquisition matrix, using a slice-selective spin echo sequence, with one slice, two averages, and five different values of the diffusion gradient, TE = 50 ms, TR = 200 ms, *T* = 20.56 ms, and δ = 10 ms. Spatial resolution was therefore 1000 (slice thickness) × 469 × 117 μm. Extent of vasogenic edema or infarction was calculated from T2WI 3D MR images using the ParaVision 3.0.1 (Bruker, Ettlingen, Germany) software. In T2WI MR images, vasogenic edema appeared hyperintense. Addition of all the hyperintensive areas in all serial slices yielded an estimation of the lesion volume. Results are expressed as mean ± S.E.M. Data were analyzed by regression analysis based on the mixed models procedure of the Statistical Analysis System (Proc mixed; SAS for Personal Computers, Version 8, SAS Institute, Cary, NC, USA). Analysis of the image differences between ipsilateral and contralateral striata to the ET-1 injection, were performed. *P*-values lower than 0.05 were considered statistically significant.

### Rodent neurosphere culture, neurosphere differentiation, and grafting

Striatum from C57BL/6 mice, transgenic for the GFP gene [strain C57BL/6-TgN(ACTbEGFP)1Osb] (Okabe et al., 1997) were used to obtain NSCs constitutively expressing GFP as previously reported (Doncel-Pérez et al., 2009) (step 1). Neurospheres, isolated from E13 striatum, were incubated in NB27 medium (step 2), a mix of Neurobasal medium and B27 supplement (GIBCO, Scotland, UK) containing human bFGF (10 ng/mL) and EGF (20 ng/ml; Peprotech, New Jersey, USA), L-glutamine (0.5 mM), L-glutamate (25 mM), penicillin (100 U/mL), streptomycin (0.1 mg/mL) (all from Sigma-Aldrich), and fungizone (2.5 g/mL; Invitrogen, Madrid, Spain). Then, neurospheres were expanded in the same incubation medium (step 3) and then (step 4) either dissociated by mild trypsinization for expansion or subjected to a differentiation protocol, as described below, before transplantation. Regarding neurosphere differentiation, we recently found that *in vitro* differentiation of striatal NSCs could be induced using a simple four-step method. Briefly, after formation of neurospheres (step 2), selection and expansion of ESCs was carried out in NB27 medium (step 3). Then, neurospheres were dissociated and plated (2 × 10^5^ cells/well) (step 4) into tissue culture dishes pre-coated with polylysine (PLL) and containing NB27 medium without serum or growth factors. Immature NSCs cells were allowed to differentiate for five days in a humidified atmosphere containing 95% air and 5% CO_2_ at 37°C. After five days, GFP cells were collected by mild trypsinization, suspended in Hanks Balanced Salt Solution (HBSS, Sigma) and the cell suspension adjusted to obtain an appropriate concentration of cells for injection, minutes before transplantation. NSCs obtained either by dissociation by mild trypsinization or neural-differentiated cells obtained in the step 4 above, were used for transplantation in the ischemic model.

Two hours after ET-1 intranigral injection, neurosphere cells were re-suspended in HBSS at a concentration of 20,000 cells/μl and immediately transplanted into two points at the intended area of striatal infarction: AP, 0.8; L, −4.0, and V, −5.6 mm and AP, 1.5, L, −3.0 and V, −5 mm below the dura. Each animal received 5.0 μl of grafting cell suspension or vehicle at a rate of 1 μl/min. The scalp was sutured and the animals were allowed to survive for 14 and 28 days. The animals were filmed before and 24 h after surgery to record motor performance at baseline and to demonstrate actual injury.

### Histology and immunohistochemistry (IHC)

Animals were perfused 14 days after surgery. For this, deeply anaesthetized animals (Equithesin, Janssen Laboratories, 2.5 ml/kg intraperitoneally) were ventilated and perfused through the left ventricle with PBS followed by 500 ml of a fixative solution containing 4% (w/v) paraformaldehyde in 0.1 M PB at pH 7.4. Animals for electron microscopy were perfused with the same volume of fixative solution containing 4% paraformaldehyde and 0.1% glutaraldehydde. Brains were then removed, cut into small blocks and post-fixed for 4 h at room temperature (RT) in the same fixative without glutaraldehyde. The blocks were then transferred to a 30% sucrose solution in PB and stirred at 4°C until they sank. Staining for light and electron microscopy was achieved according to the standard avidin-biotin-peroxidase complex (ABC) method (Hsu et al., 1981). Specific antibodies to neuronal nitric oxide synthase (nNOS, 1:4000, rabbit polyclonal; Martinez-Murillo et al., 2007) were used to analyze the neuroprotective effect of NSCs. Antibodies to tyrosine hydroxylase (TH, 1:100, mouse monoclonal; Martinez-Murillo et al., 1988) were used to analyze the effect of vasoconstriction and NSCs transplant on striatal dopamine protein expression. The NSCs integration and migration were evident with GFP antibodies (1:1000, rabbit polyclonal serum, Invitrogen-Molecular Probes). β-Tubulin III (1:1000, mouse monoclonal, Sigma-Aldrich) was used as a neuronal marker. To assess the proliferation of the transplanted cells *in vivo* and exclude any tumor formation by transplanted cells, we used specific antibodies against bromodeoxyuridine (BrdU, 1:1000, monoclonal anti Rat, Abcam), doublecortin (1:1000, guinea pig polyclonal serum, Chemicon), phospho-Histone 3 (p-H3, 1:400, rabbit polyclonal IgG, Upstate Biotechnology) and nestin (1:1000, rabbit polyclonal anti human IgG, Urban Lendahl). In order to evaluate cell differentiation, we used antibodies against calbindin (CB D28k, 1:3000, polyclonal rabbit antiserum, Millipore), choline acetyltransferase [ChAT, monoclonal rat antibody, (Martinez-Murillo et al., 1990)], somatostatin [SOM, 1:50, rat monoclonal antibody YC7, (Ribeiro-da-Silva and Cuello, 1990)], parvalbumin (1:500, monoclonal mouse antibody, Sigma), and calretinin (1:200, polyclonal goat antiserum, Chemicon). The glial scar was evident by using tomato lectin (TL-Sigma) and antibodies to glial fibrillary acid protein (GFAP, 1:500, mouse monoclonal antibody, Chemicon). Angiogenesis and vascular endothelial cells were evaluated using antibodies to CD31 (10 μg/ml, mouse monoclonal antibody, P2B1-Abcam) and collagen type IV (1:100, rabbit polyclonal, Fitzgerald). Pepsin pretreatment was used prior to collagen immunostaining (Franciosi et al., 2007), by incubating sections in 1 mg/ml pepsin in 0.2 M HCl, for 10 min at 37°C followed by thorough washing of the sections.

#### Light microscopy IHC

After fixation and cryoprotection, serial 40 μm thick coronal frozen sections were cut with a 2800 Frigocut (Reichert-Jung) microtome and free-floating sections processed for immunocytochemistry, using antibodies diluted in PBS containing 0.2% Triton X-100. After incubation with the specific primary antibody, the sections were thoroughly washed with PBS and then incubated with the corresponding biotinylated secondary antibodies, i.e.: goat anti-rabbit, anti-rat, anti-mouse IgGs or anti-guinea pig IgGs and rabbit anti-goat IgGs (1:100, Vector Laboratories). After additional washes, sections were incubated with peroxidase-linked ABC (Vector Laboratories) for 90 min at RT. To reveal the peroxidase activity, tissue sections were pre-incubated with 0.06% 3,3′-diaminobenzidine tetrahydrochloride (DAB) (Sigma Co) in PBS for 10 min at RT and afterwards in the same solution containing 0.03% H_2_O_2_. The DAB reaction was interrupted at times chosen by inspection of trial sections (approximately 5 min). Control sections were routinely processed by either omitting the primary antibody or replacing it by an equivalent concentration of rabbit, rat or mouse IgGs. After the DAB reaction, sections were washed in PBS and mounted on glass slides coated with 0.5% gelatin in 0.05% chromium potassium sulphate. Sections were then dried, cleared in xylene and covered with Dammar resin (Merck). DAB photomicrographs were captured with a digital camera (Polaroid DMC IE, Cambridge, MA, USA) attached to a bright field microscope (Zeiss Axioplan 2, Jena, Germany).

#### Immunofluorescence IHC

Alternatively, GFP-cells were plated (2 × 10^5^ cells/well) on PLL pre-coated glass coverslips and incubated 5 days in NB27 medium without growth factors, for cell differentiation and immunocytochemistry. The cells on PLL-coated glass coverslips were fixed in 2% paraformaldehyde/sucrose in PBS (12 min, 25°C), washed with PBS and immunostained was initiated with a pre-incubation for 30 min, at 25°C, in PBS containing 0.1% Triton X-100. NSCs were then incubated (16 h at 4°C) in the same solution containing the specific primary antibodies mentioned above. After repeated washing with PBS, primary antibodies were visualized using the corresponding Alexa-conjugated secondary antibodies, e.g., Alexa Fluor® 488 donkey anti-rabbit, Alexa Fluor® 594 donkey anti-mouse and Alexa Fluor® 568 goat anti-rat (1:1000, Invitrogen, Molecular Probes) for 2 h at RT and mounted on glass slides with anti-photobleaching mounting media (DABCO/Mowiol) (Gomez et al., 2003). For control experiments, slices were stained with secondary antibodies only. Sections were counterstained with 4,6-diamidino-2-phenylindole (DAPI, 300 nM in PBS, Invitrogen) and examined on a LEICA TCS SP5 scanner confocal microscope (Leica Microsystems GmbH, Wetzlar, Germany). For control experiments, slices were stained with secondary antibodies only.

#### Electron microscopy (EM) IHC

The distribution of GFP immunostaining at the ultrastructural level was performed to elucidate the fine morphology and integration of pluripotent NSCs into the surrounding tissue of the host, within the boundaries of the striatum 14 days after the implant. The EM procedure followed in this study was the same as previously described (Martinez-Murillo et al., 1990). Pre-embedding immunocytochemistry was performed on six ET-1 injured animals, four transplanted and two sham operated. Briefly, cryoprotected tissue blocks were rapidly frozen with liquid nitrogen and thawed in cold 0.1 M PB to improve the antibody penetration (Eldred et al., 1983). Sections (40/μm thick) were cut with a Vibratome (Leica Microsystems GmbH, Wetzlar, Germany) and immunostained, either for GFP or GFAP or TH, as for light microscopy except that Triton X-100 was not included in the incubation solutions. After DAB reaction, the sections were washed with PBS (1 h), postfixed in 1% osmium tetroxide in 0.1 M PB, pH 7.4 (1 h), dehydrated in graded ethanols (1% uranyl acetate was included at the 70% ethanol stage for 30 min), mounted on Durcupan ACM resin (Fluka) slides under a plastic coverslip and cured for three days at 56°C. Selected areas of the caudate-putamen bearing stained structures were dissected out, re-embedded in Durcupan in plastic capsules and semithin sections (2–2.5/μm thick) were then prepared. Selected semithin sections were re-embedded in Durcupan in plastic capsules. Ultrathin serial sections were then cut, mounted on Formvar-coated grids, stained with lead citrate and examined in a Jeol 1200 EX electron microscope. Controls for immunocytochemistry were prepared as previously reported (Burry, 2010). Nonspecific staining was avoided by careful selection, under the light microscope, of the immunostained semithin sections. Controls for the immunocytochemical localization of various antigens studied were consistently negative.

### Identification of dividing cells

To assess ongoing cell proliferation and exclude potential hazards such as tumor formation secondary to proliferation of the transplanted cells or the development of cells of other lineages, each rat was injected with BrdU. Subsequently BrdU bearing cells were examined with fluorescence IHC. For this, rats received two intraperitoneal injections of BrdU (Sigma-Aldrich) at a dose of 100 mg/kg (ip, diluted in saline). Injections were administered 12 h apart, starting 24 h (*n* = 3) or 48 h (*n* = 3) after 12 days of ET-1 intranigral injection. Experimental animals were killed at 24 h after the last BrdU injection. It was necessary to denature the DNA after blocking peroxidase activity. DNA denaturation was performed as previously reported (Darmopil et al., 2008).

### Image and statistical analysis

To evaluate the potential benefits of NSCs for treating the neurodegenerative effect of ET-1 injection, we chose to quantify immunoreactivity (IR) to nNOS by using an image analysis system (AIS, Imaging Research Inc., Linton, England) as reported earlier (Alonso et al., 2002b). Measurements were made in four animals per experimental group, in two independent experiments, with two independent observers to examine whether they reached the same conclusions (Darmopil et al., 2008). Four serial coronal sections per animal, through the same AP level, were processed in parallel for immunocytochemistry and examined under identical lighting and sensing conditions. In every single case, ten separate fields were chosen at random in every slice and the following parameters were measured: (a) the total number of nNOS-positive cells; (b) the fraction of nNOS-positive cells exhibiting at least one stained neurite, defined as a cytoplasmic extension process longer than twice the soma diameter (longer than 150 μm); (c) the fraction of nNOS-positive neurons without any process; (d) the immunoreative cell density expressed as the proportional area occupied by immunopositive labeling (mean ± standard error) and the distribution of neurite-length populations expressed as percentage ± standard error. The statistical analysis was performed with Sigma Stat Version 2 program and statistical significance level was set at *p* < 0.05.

### Neurological scoring

Once pre-operative weight was regained for more than 2 days, animals were housed together as one group. Rats (ET-1, *n* = 4 and ET-1 + NSCs, *n* = 4) were assessed for recovery of neurological function by a blind observer at 24 h and at 3, 7, 14, and 28 days post-surgery. The animals were filmed before and 24 h after surgery to obtain baseline and post-injury behavioral data (Bederson et al., 1986). Rats were also scored in a drug-free test of forelimb akinesia (Lundblad et al., 2002). This protocol involved placing a rat within a glass cylinder (20 cm diameter and 30 cm height) whereupon the animal was videotaped for 3 min using a digital camera (Sony Handycam HDR-SR10E). A mirror was placed behind the cylinder at an angle to enable the rater to record forelimb movements. The number of supporting paw-placements performed independently with the left and the right paw were counted. Only wall contacts where the animal supported its bodyweight on the paw with extended digits were counted. A lower limit of 20 wall contacts per session was set to ensure the sensitivity of the test. An asymmetrical limb use score was computed by normalizing the number of wall contacts made with the forelimb contralateral to the lesion (left) as a percentage of the total number of wall contacts. Following behavioral analysis, the animal was killed to enable tissue collection and IHC for confirmation of lesion. Statistical analysis was performed with a commercial program (Sigma Stat Version 2), specifically with the Mann-Whitney Rank Sum Test ANOVA for the forelimb akinesia analysis.

## Results

Rats were subjected to focal brain ischemia following stereotaxic injection of ET-1into the right SN. Two hours after ET-1 intranigral injection, a group of animals received an injection of 5.0 μl of neurosphere cells in HBSS (20,000 cells/μl) into a rostral (AP, 1.5, L, −3.0 and V, −5.0 mm) and caudal (AP, 0.8; L, −4.0, and V, −5.6 mm) area of the striatal infarction site, with the aim to cover the both the ventrolateral and dorsolateral zones which are normally affected by experimental lesions that alter the control of forepaw adjusting steps (Chang et al., 1999). After NSCs treatment, the animals were evaluated at 24 h and at 2, 3, 7, 14, and 28 days after injury. In general a better efficacy of cell transplantion was obtained by using neural-differentiated cells generated from immature NSCs, which were allowed to differentiate for five days and which were then collected by mild trypsinization.

### Ischemia model

**Figure 1:**
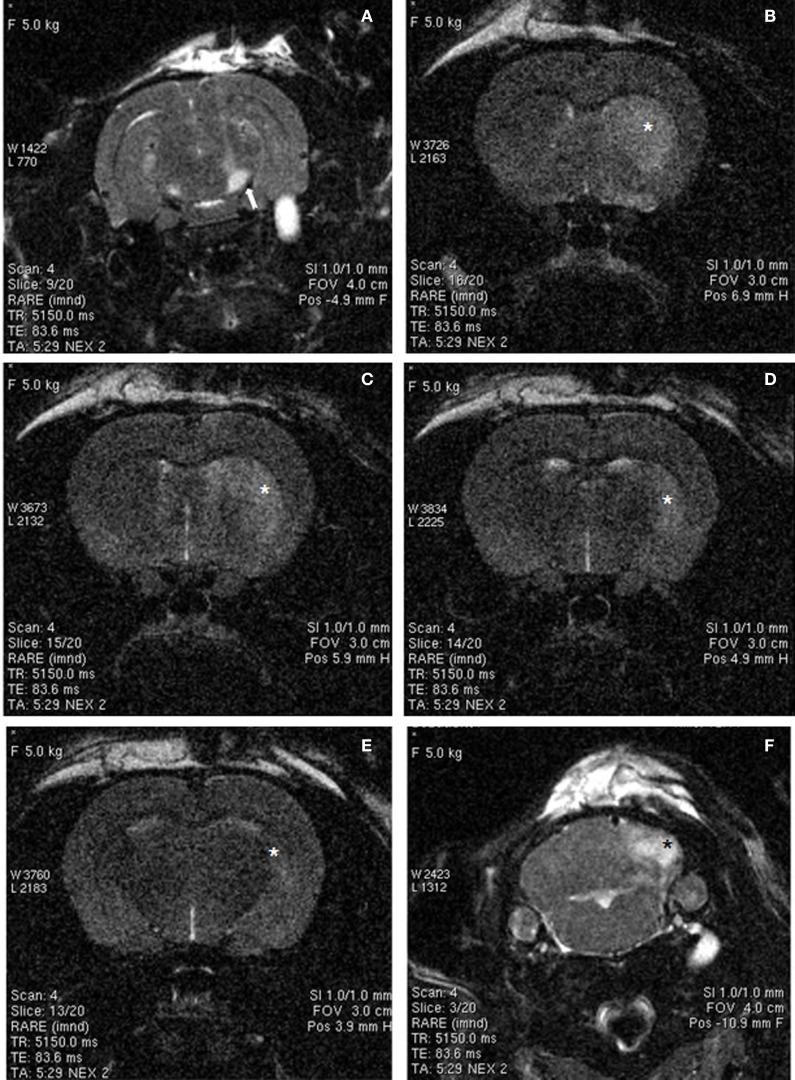
**MRI images of rat brain 48 h after ischemia, illustrating coronal T2-weighted scans at different levels of the basal ganglia (A–E) and cerebellum (F).** Increased signal intensity (arrow) can be observed in the ET-1 injected right SN **(A)**. In addition, hyperintensive areas (asterisks) were observed in the striatum **(B–E)** and cerebellum **(F)**, suggesting small-vessel ischemic disease. Widespread hyperintense areas in the right brain also suggest extensive infarcts, whereas contralateral brain areas were unaffected.

**Figure 2:**
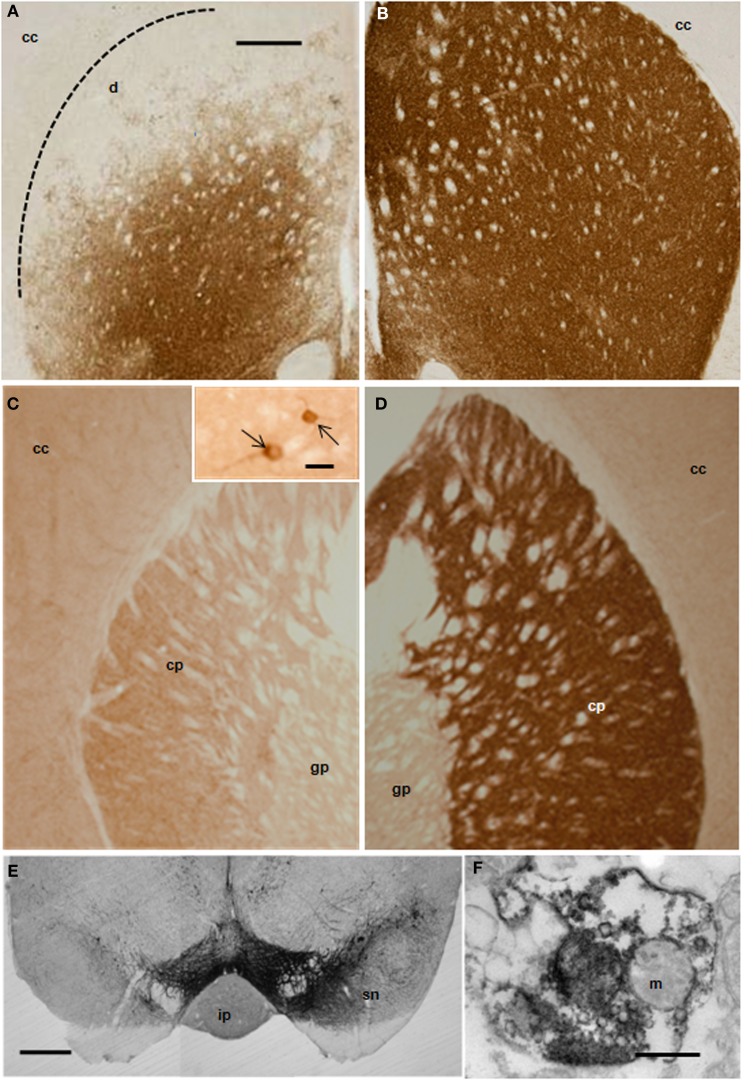
**Distribution of TH-IR in the rat striatum after ET-1 injection into the right SN.** Photomicrographs **(A–E)** represent coronal brain sections from representative animals with unilateral ET-1 SN injection at 2 **(A,B)** and 7 days **(C,D)**. Extensive unilateral DA depletion of the nigro-striatal pathway was achieved reliably in mice with a single stereotaxic ET-1 injection into the SN **(E)**. Striatal dopamine was depleted in areas unilateral to the injection within the striatum **(A,C)** and SN **(E, left hand side)**, when compared to the contralateral side **(B,D and E right hand side)**. Inset in **(C)** illustrates DA neurons (pointed by arrows) within the homolateral striatum to the injection. **(F)** electron micrograph illustrating a TH-immunopositive nerve ending showing signs of degeneration within the homolateral striatum. Scale bars: 500 μm **(A–E)**; 0.5 μm **(F)**; 20 μm inset in **(C)**. Abbreviations - cc: cerebral cortex; cp: caudate-putamen; gp: globus pallidus; ip: interpeduncular nucleus; m: mitochondria; sn: susbtantia nigra.

Ischemic damage was evaluated 48 h after the intranigral injection of ET-1 by both MRI (Figure 1) and TTC staining. Ischemic lesion images obtained by MRI closely paralleled TTC staining. The injection of ET-1 produced a lacunar infarction limited to the nigrostriatal pathway. MRI showed a significant increase in the apparent diffusion coefficient (ADC) in the injected SN (Figure 1A), which represented ischemia-induced vasogenic edema. The corresponding striatum also showed increased ADC from rostral to caudal levels (Figures 1B–D), reflecting a broad vascular affectation within these areas (Brubaker et al., 2005). The ADC values in the contralateral striatum remained at the same levels as in sham operated rats across the different time periods. Increased ADC values were also detected in the homolateral posterior lobule of the cerebellum (Figure 1F). Characteristically, adjacent regions to ischemic striatal areas, including the cerebral cortex, globus pallidus, and thalamus, lacked signs of vascular affectation (Figures 1A–E), suggesting that lacunar infarction was limited to the nigrostriatal pathway and cerebellum. Because implanted NSCs were confined to the striatum, the study of the histological effects was focused at this level. Taking into account that TH is the hallmark of DA structures within the striatum, we studied the expression of TH by immunostaining in the ischemic model (Figure 2) to evaluate the effect of ET-1 injection on striatal dopamine. Two days after ET-1 injection, IHC revealed that extensive striatal areas ipsilateral to the intranigral ET-1 injection, were devoid of TH fibers (Figure 2A), in stark contrast to the contralateral side (Figure 2B). The lack of TH-immunostaining affected mainly the dorsolateral striatum and initially seemed to omit ventromedial DA afferents (Figure 2A). This is consistent with a topographic preservation of fibres from the SN, since the injection site was restricted to the lateral half of this structure and it is known that the nigrostriatal projection is topographically organized (Faull and Mehler, 1978). One week after ET-1 injection, the lack of TH-positive fibres progressed, affecting almost the total area of the striatum at all dorsoventral levels (Figure 2C). In marked contrast, the contralateral side remained unaffected (Figure 2D). Characteristically, striatal regions devoid of TH fibers exhibited intensely labeled TH–positive cells (Figures 2C, 5K). These cells were observed regardless the time elapsed since the ET-1 injection, being present early when dopamine depletion was evident within the dorsal striatal regions. The results also indicate a relative resistance of these cells to ischemia damage. Overall lack of TH-immunostaining correlated well with MRI (Figures 1B–D). Previous reports have shown that TH-positive cells may be projection neurons (Darmopil et al., 2008). The lack of DA innervation in the striatum is consistent with a dramatic loss of expression of TH in DA neurons in the SN (Figure 2E). At the EM level, TH-positive fibers in the striatum that appear floating in a lake of amorphous edema fluid exhibited striking signs of degeneration with crumpling of synaptic vesicles and clear mitochondria with fragmented cristae (Figure 2F).

**Figure 3:**
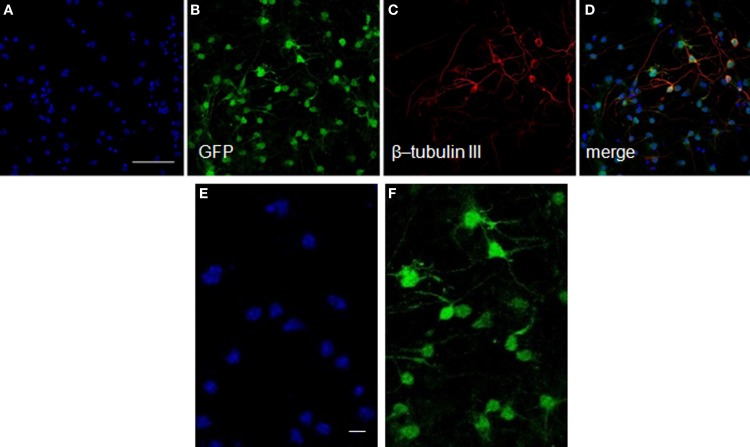
**Neurons from neurosphere cells isolated from the E13 striatum of GFP transgenic mice.** The GFP-mouse neurospheres were dissociated to individual cells, plated over an adhesive substrate and incubated for five days in the absence of serum or growth factors. The GFP rodent cells visualized in green **(B)** showed a neuronal morphology and expressed high levels of β-tubulin III, an early neuronal marker detected with a monoclonal antibody **(red, C,D)**. The nuclei **(A,D)** revealed by Hoechst staining are shown in blue. **(E)** and **(F)** represent high-power magnification photomicrographs of **(A)** and **(B)**, respectively, showing that all GFP labeled cells exhibited Hoechst staining. Scale bars: 50 μm **(A–D)**; 20 μm **(E,F)**.

After transplantation into the brain, NSCs progeny are confronted with multiple fate decisions, including survival, death, proliferation, migration, or differentiation into a specific lineage. We have shown in culture that neurosphere-derived cells are able to differentiate into a type of axonal-growth-promoting ensheathing glia, named aldynoglia (Gudino-Cabrera and Nieto-Sampedro, 2000; Doncel-Pérez et al., 2009). As such glia and neurons positive for GFP expression (Figure 3) facilitated the tracking of transplanted cells into the brain. The capacity of these cells to produce neurons and astrocytes was also analyzed. After five days of incubation in defined medium, a subset of cells derived from neurospheres assumed a neuronal-like shape (Figure 3). These cells showed global expression for GFP (Figure 3B), and a large quantity of them were also positive for β-tubulin III, a specific pan-neuronal marker (Figures 3C,D).

### Survival and differentiation of grafted NSCs

**Figure 4:**
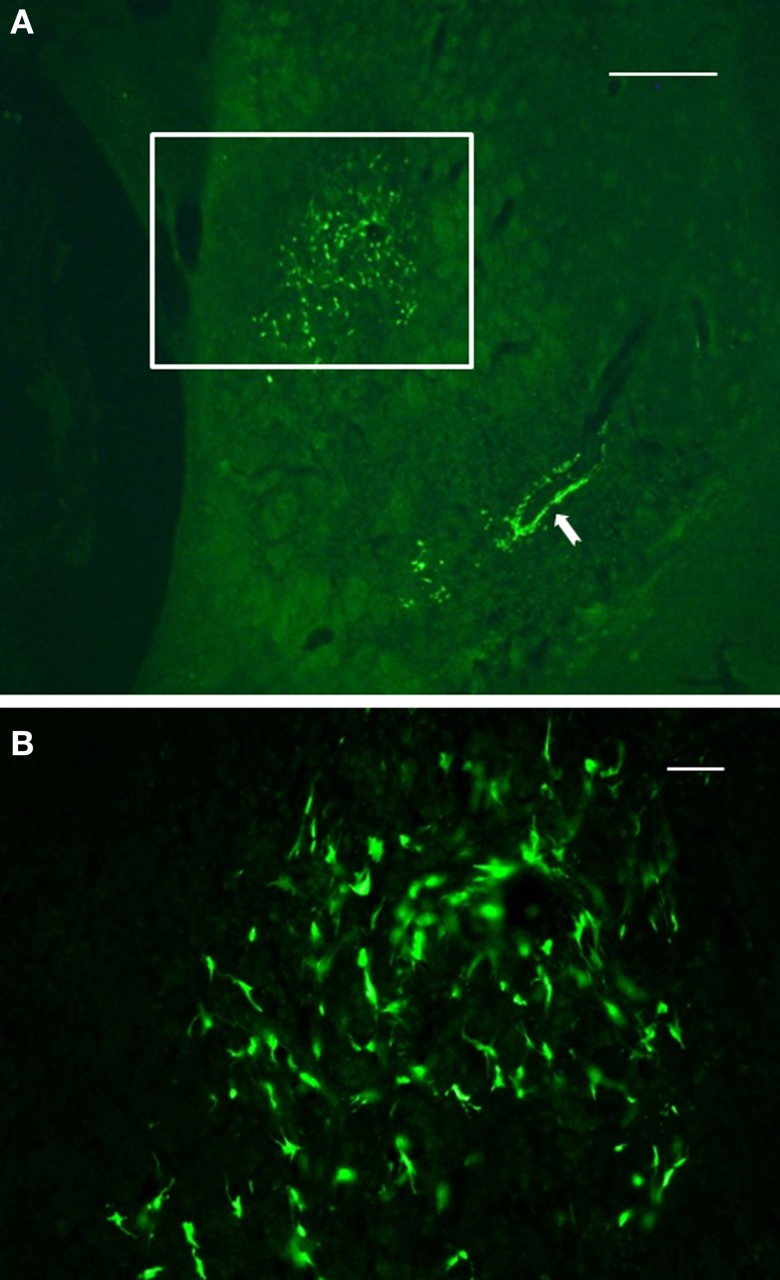
**Immunofluorescence photomicrographs of a coronal section through the graft site showing *in vivo* survival and morphological maturation of cells differentiated from NSCs expressing GFP, 7 days after transplantation into the ischemic striatum. (A)** reflects the area or size of striatal region affected by infarction. **(B)** represents a high-power magnification of the region of interest shown in **(A)** (boxed area). Note the preservation and clustering of NSCs at the injection site. Also note that a subset of NSCs delineated a blood vessel located some distance from the injection site (solid arrow), suggesting that these cells may migrate throughout the infarct area from the initial injection site. Scale bars: 500 μm **(A)**; 30 μm **(B)**.

Final results of NSCs transplantation include data from *in vivo* MRI, TTC staining, immunocytochemistry and behavior. Following 48 h after administration of ET-1, morphological signs of stroke were stable up to 14 days post injection. The size of the infarction was expressed as percent area of the striatum in coronal sections. All rats with ET-1 injection showed large areas of infarction greater than 80% of the striatum. The distribution of the infarcted areas in transplanted striatum was more varied and generally did not include the area of transplant (Figure 4). The size of the infarcted area per lesioned group was 90 ± 11% (mean ± S.E.), which was significantly greater than the average size of infarction for the transplanted rats (61 ± 9%, *p* < 0.05).

**Figure 5:**
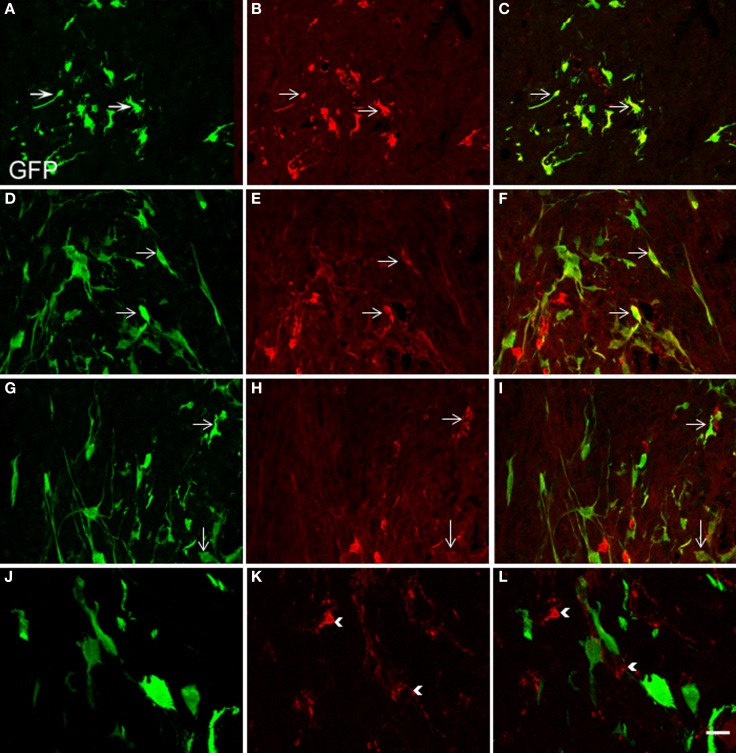
**Confocal laser-scanning microphotographs taken from coronal histological sections through the NSCs transplanted striatum.** Histological sections with GFP labeled cells **(green, A,D,G,J)** were immunostained for ChAT, SOM, calbindin and TH antigen IHC **(red, B,E,H,K, respectively)**. Corresponding double immunostaining is indicated **(C,F,I,L)**. Note in the merged confocal microscopy images, that several neural GFP elements colocalize with ChAT **(A–C)**, SOM **(D–F)**, or calbindin antigens **(G–I)**. Colocalization is shown in yellow. However GFP-positive structures consistently lacked TH immunostaining **(J–L)**. Scale bar: 50 μm **(A–I)**; 40 μm **(J–L).**

**Figure 6:**
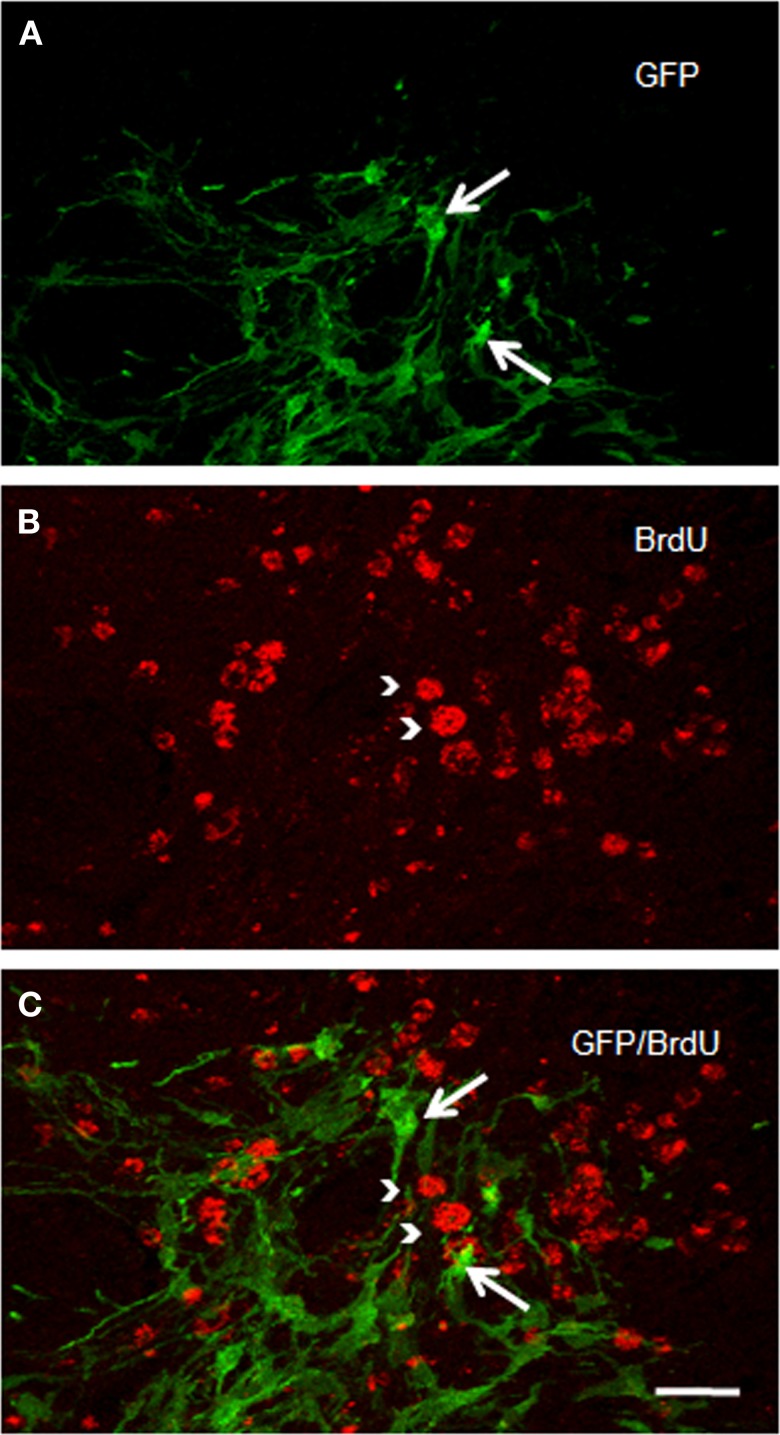
**Confocal laser photomicrographs taken from coronal sections through the grafted striatum of a rat that received two intraperitoneal BrdU injections (at an interval of 12 h), 14 days after ET-1 intranigral injection.** Experimental animals were killed 48 h after BrdU treatment. Notice in the merged confocal microscopic image **(C)** that GFP-positive structures in **(A)** do not express BrdU immunostaining as shown in **(B)**. Long arrows indicate GFP-reactive cells. Arrow heads point to BrdU-positive structures. Scale bar: 40 μm.

**Figure 7:**
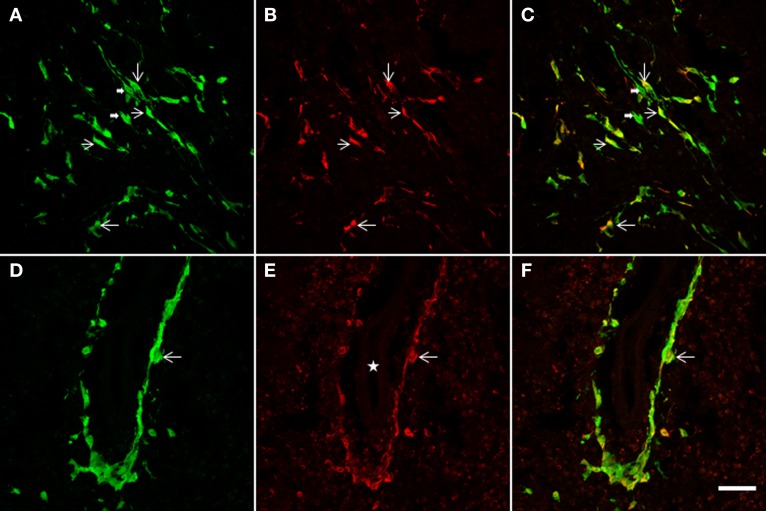
**The NSCs grafted striatum, after ET-1 ischemic insult, showed colocalization of GFP with the GFAP glial marker.** Two representative horizontal planes **(A–C,D–F)** of the confocal microscope are shown, captured from coronal histological sections through the grafted striatum. In the merged images in yellow **(C,F)** long bipolar GFP cells are also shown to express GFAP **(C)**. **(D–F)** represent a high-power magnification of the Figure 4A (indicated by a white solid arrow). Note that a population of GFP-positive cells lying near the blood vessel **(D)** also exhibit GFAP **(F)**. Also note that the labeled cells do not show the hypertrophic appearance characteristic of reactive gliosis. Thin arrows indicate double labeled astrocytes and thick arrows point to single-labeled cells. The white star in **(E)** indicates the lumen of the blood vessel. Scale bar: 50 μm **(A–C)**; 40 μm **(D–F).**

Two weeks after NSCs transplantation, GFP immunostaining revealed abundant GFP-positive cells and cellular processes extending into the transplanted host (Figures 4–7). Quantitative double-fluorescence IHC carried out to determine whether GFP-positive cells expressed striatal neurotransmitter markers, showed that neuronal differentiated NSCs accounted for 26 ± 6.2% of striatal GFP-positive cells. Most GFP-bearing neurons expressed IR to ChAT (Figures 5A–C), or somatostatin (Figures 5D–F). These results were confirmed by using other commercial species-specific antibodies. No colocalization of GFP-cells with parvalbumin or calretinin was observed (data not shown). However, some GFP-positive neurons expressed calbindin (Figures 5G–I). Quantification under confocal microscopy demonstrated that 53 ± 5.3% of striatal GFP-immunoreactive neurons also expressed ChAT-IR, 45 ± 4.3% colocalized with somatostatin-IR and 3 ± 0.9% expressed calbindin-IR. Thus, as detected by IHC, GFP-derived neurons displayed mostly the phenotype of striatal interneurons. In spite of the fact that many TH-positive cells were detected within the dopamine denervated striatum (Figures 2A,D, 5K), no colocalization was noted with GFP-reactive cells (Figures 5J–L). Characteristically, the presence of the transplant did not alter the TH-fiber innervation or the TH-cell number.

We checked whether NSCs are still proliferating. Numerous BrdU-positive cells were identified in the ischemic striatum of transplanted rats examined two weeks after ET-1 injection (Figure 6). BrdU-positive cells were homogeneously distributed in the striatum; but BrdU labeling never overlapped with GFP immunostaining (Figure 6C), suggesting a role for a different cell population and no proliferation of NSCs. Immunostaining to detect doublecortin and p-H3, which stains migrating and differentiating neurons and mitotic cells, respectively, were negative. However, nestin-immunostaining, that labels intermediate filament protein of dividing and migrating cells, was prominent (not shown) suggesting strong gliosis throughout the injured striatum. Taken together, our IHC data suggest the maturation of GFP-positive cells and excludes the possibility of tumor generation arising from transplanted cells at the time studied. However, the possibility for a tumor to develop over a longer period after NSCs transplant cannot be ruled out.

### NSCs transplantation induces gliogenic response

In addition to a neuronal striatal differentiation of transplanted GFP cells, differentiation of NSCs to an astrocyte lineage and eventual contribution to reactive gliosis was assessed following a double immunohistochemical fluorescence staining for GFAP and GFP (Figure 7). After 2 weeks survival, quantification under confocal microscopy showed that 67 ± 7.3% of striatal GFP-positive cells also expressed GFAP. Characteristically two populations of double labeled cells were detected: either bipolar aldynoglia-like cells (Gudino-Cabrera and Nieto-Sampedro, 2000; Gomez et al., 2003; Muneton-Gomez et al., 2004; Doncel-Pérez et al., 2009) (Figures 7A–C) or astrocyte-like multipolar cells in close association with blood vessels (Figures 7D–F).

### Positive effect of NSCs transplantion on glial scar and angiogenesis

**Figure 8:**
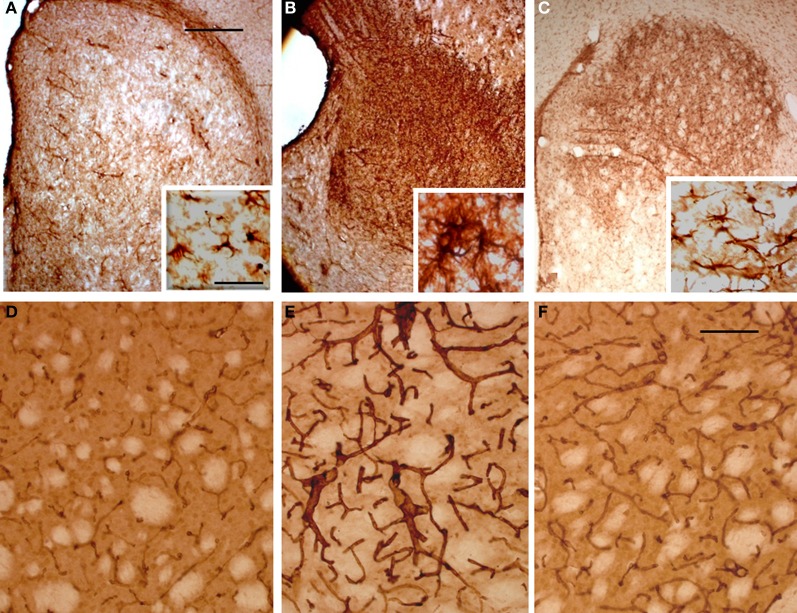
**Photomicrographs of the rat striatum 14 days after ET-injection into the right SN. (A–C)** illustrate the distribution and morphology (see insets) of GFAP-IR astrocytes within the non-lesioned contralateral **(A)**, ischemic **(B)** and NSCs grafted ischemic striatum **(C)**. The protein expression of vascular collagen at the same striatal levels is also shown **(D–F)**. Note that when panels **(D)** and **(E)** are compared an intense collagen type IV-IR is identified in **(E)** which delineates abnormal long and thick blood vessels in the ischemic striatum. Also note that the immunoreaction product in **(F)** depicts blood vessels with a similar morphology and staining intensity as **(D)**, suggesting that the NSCs implant promotes normal angiogenesis with a striking reduction of pathological capillaries. A reduction of glia hypertrophy and collagen immunoreactivity is widespread throughout the transplant site. Scale bars: 200 μm **(A–C)**; 30 μm **(D–F)**; 40 μm **(insets).**

To evaluate the glial response to ET-1 lesion and NSCs transplant, IHC was performed for GFAP. Typical astrocytes were found in all areas of the brain and similar staining was appreciated at the contralateral striatum (Figure 8A, and inset). In stark contrast, the intensity of GFAP-IR was markedly increased in the ischemic striatum. Astrocytes in this region showed an hypertrophied appearance, with long and robust processes heavily GFAP-positive (Figure 8B, and inset). After 14 days of NSCs implantation within the stroke-damaged striatum, a lower expression of GFAP IHC was observed (Figure 8C, and inset) suggesting a reduction of glial scar. Characteristically, the appearance of astrocytes in the NSCs transplanted striatum was similar to those in the contralateral side in that they exhibited a resting appearance with short and thin processes (Figure 8C, and inset), similar to the reactive astrocyte normalization after photomechanical injury with olfactory ensheating glia transplantation (Verdu et al., 2001).

In order to determine whether vascular growth was actively increased (indicating angiogenesis), a histochemical study using TL, as an effective marker of blood vessels and microglial cells, and specific antibodies to detect the endothelial cell marker CD31 and Type IV collagen, a major basement membrane component that has been implicated in the regulation of angiogenesis (Bonanno et al., 2000), was performed. TL has exclusive affinity for terminal galactosyl residues abundant in microglia and vascular endothelial cells which should be a valuable tool to examine rodent tumor angiogenesis and the ongoing immune response. The microglial response evidenced with TL showed the same pattern of labeling within all brain coronal sections with no striking differences between the contralateral or ipsilateral infarcted striatum (data not shown). In contrast the NSCs implant or vehicle injection site in sham animals produced a more pronounced staining, due to mechanical damage associated with needle injection (Gomez et al., 2003). Alteration of blood vessels ipsilateral to ET-1 injection was also observed but was masked by intense glial labeling. Therefore, an immunohistochemical study with antibodies to CD31 (not shown) and collagen type IV (Figures 8D–F) was made to evaluate angiogenesis which would indicate areas where the blood brain barrier failed to function properly. Pepsin pre-treatment for collagen type IV IHC improved the staining of blood vessels and vasculature in the striatum (Figures 8D–F). Fourteen days after ET-1 injection in the SN, the ischemic non-transplanted striatum exhibited anomalous blood vessels bearing intense collagen-IR (Figure 8E). In contrast, the transplanted ischemic striatum (Figure 8F) showed vasculature with morphology and collagen-IR similar to that of the contralateral uninjured striatum (Figure 8A). These results indicate that NSCs and their progeny might exert a neuroprotective role in the ischemic striatum by reducing astrocytic gliosis and aberrant angiogenesis. Therefore, the pathological condition resulting from abnormal angiogenesis after ischemia might be overcome by NSCs grafting, ensuring that the ischemic striatum receives an adequate blood supply.

### Neuroprotective effect of NSCs transplantation for ischemic insult

**Figure 9:**
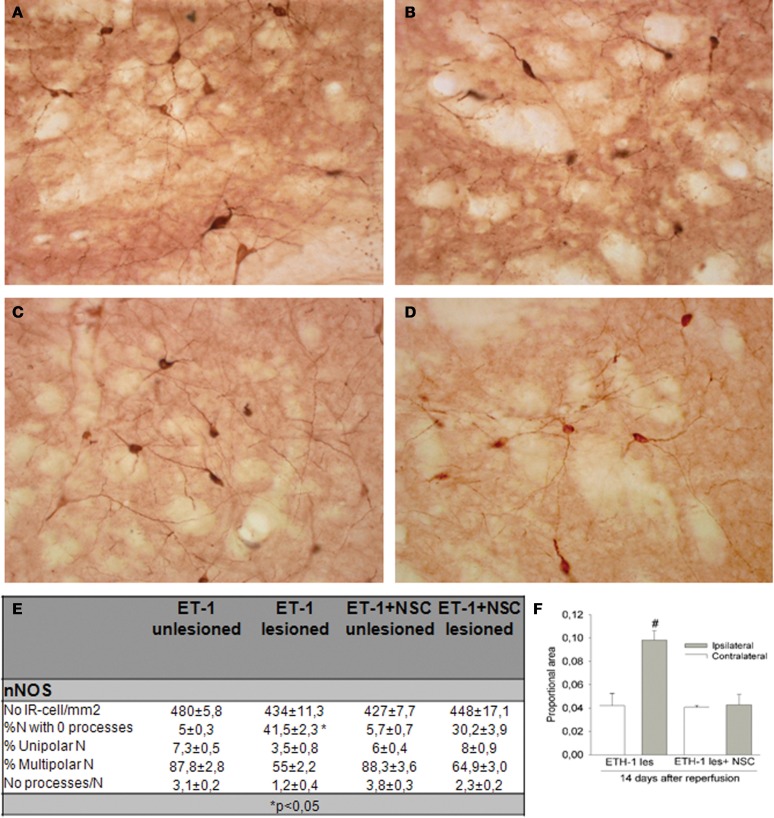
**Immunocytochemical expression of nNOS in the NSCs grafted striatum after ET-1 induced ischemic damage.** Photomicrographs were obtained from coronal tissue sections. Tissue from intranigral lesioned animals 14 days after injury showed representative positive nNOS signal in the contralateral **(A)** and ischemic striatum **(B)**, while **(C)** and **(D)** depict nNOS-positive neurons within the contralateral and ischemic striatum respectively in a NSCs-grafted rat. **(A and inset in A)** depicts multipolar nNOS neurons and processes with a normal appearance. **(B)** represents nNOS-positive neurons showing an abnormal morphology. Notice comparing insets in **(A)** and **(B)** that a reactive neuron **(inset in B)** shows soma condensations. Also notice in **(B and inset)** numerous varicose or fragmented processes in the neuropil. **(C)** represents standard nNOS-IR neurons. When photomicrographs **(A)** and **(C)** are compared, the nNOS-positive neurons are observed to exhibit a similar morphology and staining intensity. **(D)** illustrates typical nNOS-reactive cells that partially preserve a normal neuronal arborization. When **(D)** was compared with **(B)**, a lower proportion of nNOS-positive neurons (30%) showed soma condensations. **(E)** represents the distribution of cell number and neurite-length populations expressed as a percentage ± SEM (^*^p < 0.05; versus transplanted ipsilateral side; One-Way ANOVA). In **(F)** histograms are presented showing the proportion of stained area with nNOS-IR neurons in the striatum, expressed as a mean ± SEM (#*p* < 0.05 vs. contralateral side; One-Way ANOVA). Scale bar: 50 μm.

In this study the impact of the unilateral transplantation paradigm on the immunocytochemical expression of nNOS after ET-1 administration was evaluated (Figure 9). Fourteen days after intranigral injection of ET-1, nNOS neurons in the contralateral striatum showed a normal morphology (Figures 9A,C) and the number of these immunoreactive cells in the ipsilateral ischemic striatum was similar to that in the contralateral striatum in both non-transplanted and sham operated animals. However, staining intensity was higher within the ischemic striatum (Figure 9B) reaching twice that of the contralateral unaffected side (Figure 9F #*p* < 0.05 One-Way ANOVA). In the ischemic striatum, nNOS-IR labeled exclusively neurons with soma condensation and loss of arborizations with varicose and fragmented processes (Figure 9B). This was in contrast to nNOS-IR cells within the unaffected contralateral side (Figure 9A), where multipolar neurons exhibited 3–5 processes. The fraction of neurons without processes within the ischemic striatum was very high (41.5 ± 2.3, ^*^p < 0.05 One-Way ANOVA) and those with processes rarely had more than two (Figure 9E). It was noteworthy that the increase in nNOS immunostaining was lower in the NSCs transplanted striatum and that neuronal arborization was partially preserved (Figure 9D). The average number of neurites and the percentage of multipolar neurons were higher in the transplanted striatum compared to those in the ischemic non-transplanted site. Finally, the total number of nNOS-IR neurons by unit area was not significantly different in the contralateral unaffected striatum and that of sham operated animals. From these results it appears that ET-1 lesion modifies the neuronal morphology and increases the level of expression of nNOS, while NSCs transplantation returned injured cells shape closer to controls.

### Electron microscopy

**Figure 10:**
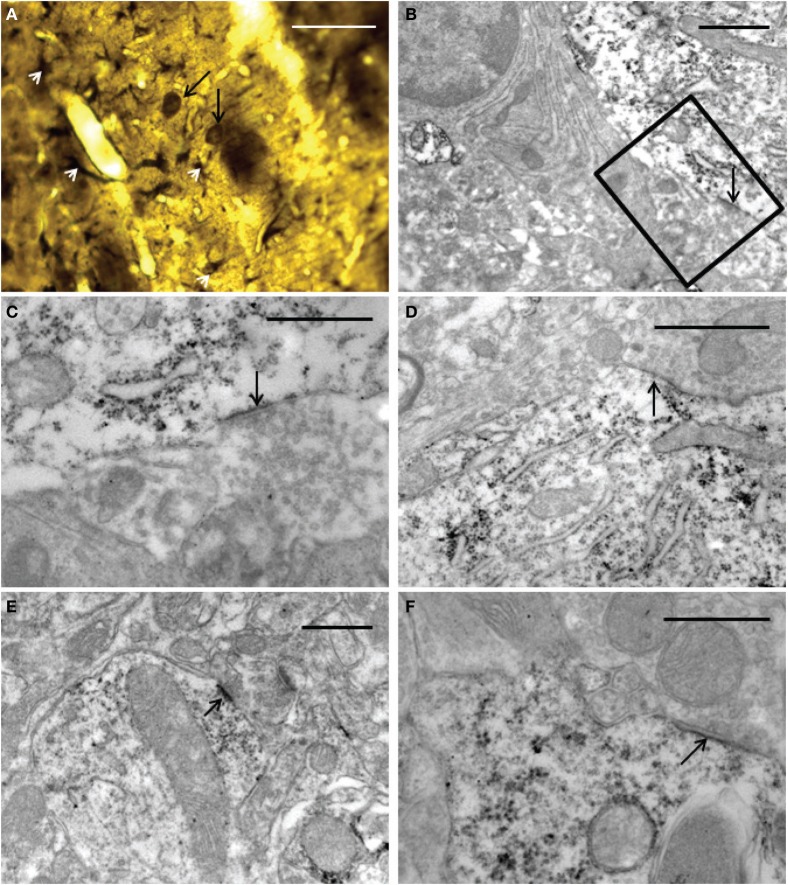
**Light (A) and electron (B–F) microscopy photomicrographs of GFP-positive structures throughout the graft core. (A)** light micrograph showing immunoreactive elements exhibiting neuronal (long black arrows) and astrocyte (white arrow-heads) morphological characteristics. **(B–F)** electron micrographs of axosomatic **(B–D)** and axodendritic **(E–F)** synaptic contacts (arrows) between unlabeled axon terminals and GFP-positive post-synaptic structures. **(C)** represents a high-power magnification showing details of the synaptic contact in **(B) (boxed area)**. Notice in **(B–F)** that the immunoreaction product is attached mainly to the rough endoplasmic reticulum and the inner face of the plasma membrane. Note also in **(E)** a typical asymmetric contact identified by the presence of subsynaptic densities. 30 μm **(A)**; 1 μm **(B,D)**; 0.5 μm **(C,E,F)**.

**Figure 11:**
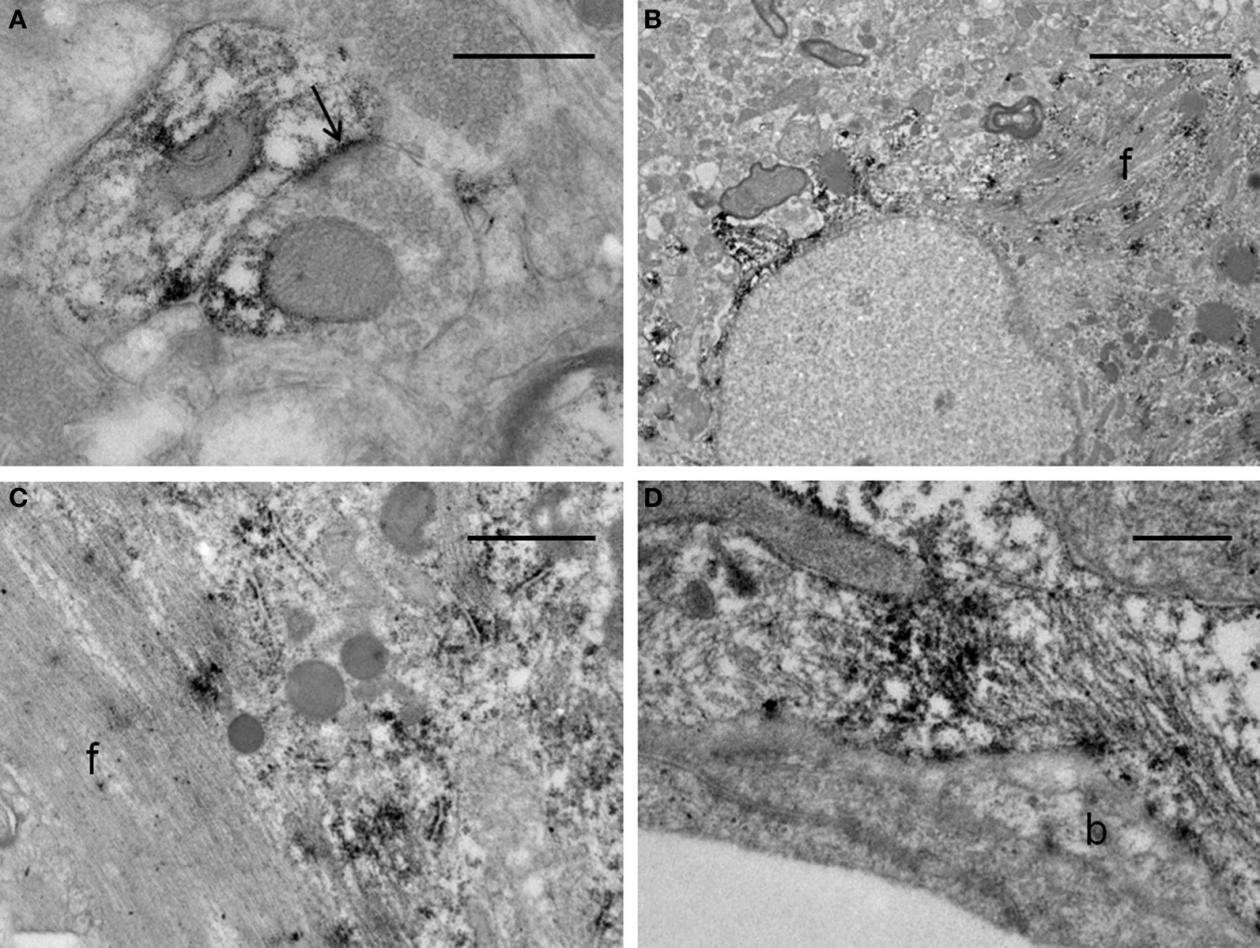
**Electron micrographs of synaptic contacts in the graft core of the ET-1 lesioned striatum, transplanted with NSCs. (A)** represents an axodendritic asymmetric synaptic contact (arrow) between labeled pre- and post-synaptic structures. **(B–D)** illustrate immunoreactive astrocytes. Notice in **(B)** that the astrocyte exhibits the aspect of a protoplasmic astrocyte with bundles of filaments (f), granules of glycogen and condensations of karyoplasm beneath the nuclear envelope. **(C)** represents a high-power magnification of an astrocyte with a bundle of filaments (f), showing deposition of the immunoreaction product associated with the rough endoplasmic reticulum. **(D)** depicts a protoplasmic astrocyte extending toward the wall of a capillary surrounding an endothelial cell. This process remains separated from the endothelial cell by the basal lamina (b). The morphology of the capillary is within normal limits. Scale bars: 0.5 μm **(A,D)**; 5 μm **(B)**; 1 μm **(C)**.

The ultrastructural analysis was carried out in selected areas of the striatum found by light microscopy to contain GFP-positive structures (Figure 10A). Endogenous GFP was localized by the presence of electron-dense peroxidase reaction product in neurons and glial cells (Figures 10B–F, 11). The reaction product was present mainly as an attachment to the outer surface of the rough endoplasmic reticulum, which was consistent with the GFP protein processing. The distribution of the reaction product correlated well with previous studies using GFP IHC. Neurons exhibited GFP-IR in cell bodies and dendritic shafts (Figures 10B–F). GFP-positive glial cells were identified as astrocytes (Figures 11B–D). These immunoreactive structures were embedded in numerous profiles of small-diameter unmyelinated fibres and clusters of large myelinated axons. It is noteworthy that whereas GFP-positive neuronal cell bodies were more frequently detected near the core of the cell implant, the GFP-IR astrocytes and dendritic shafts pervaded the striatum. Immunopositive neurons received scarce axosomatic synaptic contacts from unlabeled terminal axons that formed synapses of the asymmetric type (Gray's type I) (Figures 10B–D). In comparison with the somata, synaptic contacts established between immunonegative terminals and GFP-IR distal dendrites of various diameters were the most commonly detected (Figures 10E,F). The latter were mostly of the asymmetric type. Axodendritic contacts between GFP-IR structures were rare (Figure 11A). GFP-positive glial cells showed the ultrastructural appearance of protoplasmic astrocytes (Figures 11B,C). The nucleus of these cells exhibited a homogeneous karyoplasm surrounded by a thin rim cytoplasm, containing bundles of filaments, short GFP-positive cisternae of rough endoplasmic reticulum and common granules of glycogen. Astrocytic perivascular processes exhibiting GFP-IR were located completely covering capillaries coupled to the basal lamina (Figure 11D). As regards to GFP-immunostaining, the EM results correlated well with the results obtained with light microscopy.

### Assessment of functional outcome

Rats were allowed to move freely during the behavioral tests, specifically for observation of forelimb placement and circling behavior. Animals were held gently by the tail, suspended above the floor and observed for forelimb and trunk flexion movement (Bederson et al., 1986). Uninjured animals were moved in random directions using all limbs and stretching their body while walking. After attaching the tail and raise above the ground, rats extended normally both forelimbs towards the floor with any preference for forelimb support. In contrast, following ET-1 injections the animals moved with severe placing deficits, characterized as forelimb flexion and lateral deviation of the trunk, rotational jumps and occasionally loss of postural support. In addition, rats showed circular locomotion toward the contralateral side of the ET-1 injection. Spontaneous motor behavior decreased progressively up to the third day post-lesion.

**Figure 12:**
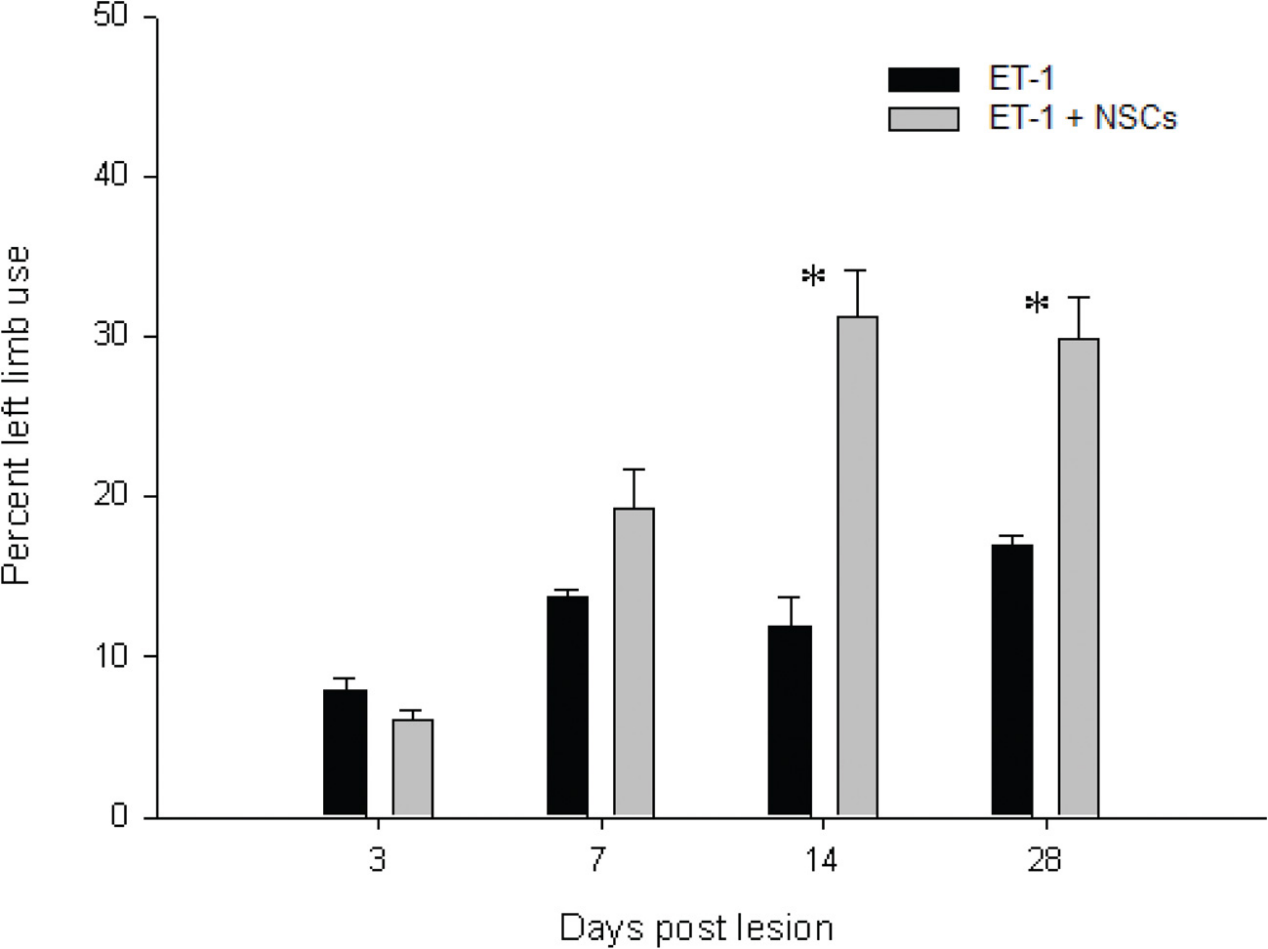
**Time course of the drug-free forelimb akinesia test.** Antiakinetic effect of NSCs transplant was scored as a percentage of contralateral limb use, in animals administered with ET-1 into the SN. The effect became apparent after 14 days and up to 28 days after injury. Data are mean ± SEM, *n* = 4 for each group (^*^p < 0.05). Forelimb asymmetry scores were normalized to the total forelimb use.

In general the animals responded variably to the novel cylinder environment, by exhibiting vertical wall exploration by standing on their hindlimbs and leaning against the cylinder with their forelimbs. Imbalance for torsional trunk movement and spontaneous rotation were observed only during the first week after injury. The time course for asymmetrical forelimb-use was evaluated from 3 to 28 days after ET-1 injection in both the untreated and NSCs groups (Figure 12). No significant difference in the number of wall contacts performed with the right limb (unaffected side) was identified when compared between groups during the testing sessions. The untreated ET-1 group relied primarily on the ipsilateral forelimb for wall support and lateral movements. In contrast the ET-1 + NSCs group gradually exhibited an increase in the percentage use of the contralateral limb, so that after 14 days post injury significant differences were observed between the experimental groups. In the untreated ET-1 group the percentage use of supporting wall contacts performed with the limb contralateral to the lesion (left) increased from 7% on the third day to 16% 28 days after injury. In contrast, the ET-1 + NSCs transplanted group revealed an improvement of the asymmetrical forelimb use score from 6 to 30% during the same period time (*p* < 0.05, limb performance vs. non-transplanted group). The degree of DA denervation was confirmed by TH IHC for each animal within each experimental group. In each case, postmortem TH IHC confirmed clear DA denervation. Therefore, we might rule out the possibility that spontaneous motor behavior after day three post-lesion could be induced by physical damage caused by the injection.

## Discussion

The mechanisms essential for cerebral plasticity, including neurogenesis and angiogenesis, are associated with repair processes, mechanisms that should be therapeutically promoted following stroke. Animal models of stroke can be divided into two main categories, depending on whether they achieve global or focal brain ischemia. In humans, global brain ischemia occurs following cardiac arrest, but the most common strokes are due to localized infarcts (Back and Schuler, 2004). In the present report we implemented a lacunae ischemic cerebral vasoconstriction model following local ET-1 administration into the SN. The resulting ischemic lesion involved the distribution area of the brain perforating arteries which produced nigrostriatal alterations as identified by IHC and MRI after 48 h of ET-1 intranigral injection and was stable during the period of study. The ischemic lesion was accompanied by extensive and progressive DA denervation that involved the SN and both matrix and striosomal compartments of the entire rostro-caudal axis of the striatum. Our results with regard to the capacity of NSCs to integrate into the host striatum are consistent with previous studies using murine or human NSCs in rodent models of stroke (Buhnemann et al., 2006) and in a Huntington's disease model (McBride et al., 2004). In a model of striatal ischemia with endothelin-induced middle cerebral artery occlusion, neural differentiation of transplanted NSCs was shown for up to 12 weeks after grafting (Buhnemann et al., 2006). Characteristically, in our model, we found detailed morphological signs of integration as early as 14 days following NSCs grafting.

Targeting functional rehabilitation and neurorepair using a cellular therapy is efficacious in both experimental and clinical studies (Lindvall and Kokaia, 2005, 2006; Locatelli et al., 2009; Darsalia et al., 2010; Lindvall and Kokaia, 2010). Stem cells derived from various sources and their neural derivatives are attractive for use in regenerative medicine to treat functional impairment resulting from traumatic injury or neurodegenerative diseases, including stroke. This strategy includes the use of NSCs derived from neurogenic zones of the developing brain (Gage, 2000; Taupin, 2006). Initially stem cells were proposed to act as a “cell replacement” mechanism, but this cell therapy may also work by providing trophic support to the injured tissue, fostering both neurogenesis and angiogenesis (Bersano et al., 2010), and thus facilitating functional recovery.

The concept of vascular Parkinsonism (VP) has developed since its description by Macdonald Critchley in 1929 (Critchley, 1929). Although the exact etiology is unknown, VP is usually secondary to microvascular occlusive episodes typically localized to basal ganglia, including SN and striatum (Akyol et al., 2006; Vaamonde et al., 2007; Orta Daniel and Ulises, 2008). Vasospasm has been considered as a possible precipitating cause of the disease (Lammie, 2000). In fact, altered expression of the ET system observed within the lacunae (Nie and Olsson, 1996) was the rationale for the development of the lacunar stroke model based on the administration of ET-1 into the SN. We have previously used a model of focal brain ischemia by the direct injection of ET-1 into the striatum with good reproducibility (Martinez-Murillo et al., 2007, 2009).

A variety of translational animal models of stroke have been developed (Bailey et al., 2009), but with difficulties for VP. Our model mimics features of human unilateral VP.

### NSCs transplanted into the striatum develop into neural cell populations

The progressive accumulation of synaptic vesicles at the presynaptic site is considered as a hallmark of synapse maturation (Blue and Parnavelas, 1983). The observation of vesicle accumulation at the presynaptic site in synaptic contacts onto GFP-positive structures suggests that NSCs-derived neurons establishes functional relationships with the neighboring structures of the host, which may be indicative of their involvement in physiological processes. The latter agrees with a previous study (Buhnemann et al., 2006). Grafted striatal NSCs generated cells with phenotype of non-DA striatal interneurons, particularly ChAT and somatostatin interneurons, and a low percentage of calbindin immunopositive cells characterized by a strong protein expression and 2–3 sparsely-branched processes, a characteristic of calbindin interneurons (Cicchetti et al., 2000).

Intrinsic striatal DA-neurons after stroke was evident in this study. This is consistent with previous reports that DA-like neurons are intrinsic to the human striatum (Cossette et al., 2005) and that the number increases markedly in animal models of PD (Huot and Parent, 2007). We hypothesize that following stroke a subset of intrinsic striatal neurons recover DA expression, as it has been reported during development (Busceti et al., 2008), but are not newly generated cells as they do not incorporate BrdU in either our study or previous reports (Darmopil et al., 2008). It appears that the DA cell population of the striatum responds either to stroke or dopamine denervation by increasing their number, possibly to compensate the loss of extrinsic DA innervation (Betarbet et al., 1997).

One major concern related to the future clinical use of intracerebral transplantation of NSCs in the damaged human brain is the potential for these self-renewing cells to continue proliferation. The incorporation of BrdU into the nucleus of dividing cells was used to demonstrate this possibility in the graft. In our study, GFP-positive cells consistently lacked BrdU immunostaining. Previous reports showed that after 1 month of differentiation conditions in an *in vitro* culture system, nearly 10% of the total cell population of striatal NSCs retained proliferative activity (Kallur et al., 2006). *In vivo* results in this study show that GFP-positive cells consistently lacked staining following BrdU experiments. In addition these labeled cells were negative for doublecortin and pH3 immunocytochemistry, suggesting that after 14 days of cell grafting GFP-positive cells acquire a differentiated, mature, phenotype. Our results are also similar to a previous study that hypothesized that the initial stroke environment, from 1 to 2 weeks after the onset of insult, promotes either differentiation or quiescence of grafted NSCs (Darsalia et al., 2007). Consistent with this finding, no teratomas were observed in animals that had received NSCs grafts. The BrdU-positive cells lacking GFP-staining reported here may represent neuronal replacement from endogenous brain precursor cells during recovery after stroke. Such cells have been found to migrate into the damaged area of the striatum following stroke (Arvidsson et al., 2002).

### Angiogenesis prompted by NSCs transplantation

Activation of angiogenesis is a normal mechanism in response to injury (Greenberg and Jin, 2005). In our study, the number of blood vessels in the grafted striatum exceeded that in the contralateral side and in sham operated animals, suggesting that implanted NSCs and their progeny might activate angiogenesis. A previous study using NSCs, abundantly generated from E15 rat or E13 mouse corpus striatum, demonstrates that neurospheres differentiate to aldynoglia-like cells *in vitro* (Doncel-Pérez et al., 2009), which are increasingly recognized as a specific glial phenotype. Aldynoglia are similar to radial glia at an early stage of ontogenic development from NSCs to their neuronal or glial destiny (Gudino-Cabrera and Nieto-Sampedro, 2000). In our study a number of GFP-positive astrocyte-like processes were associated with blood vessels in the injured striatum. This occurs for astroglia from the very earliest stages of the central nervous system development (Marin-Padilla, 1985). In stark contrast, endothelial cells lacked GFP-IR, with the speculation that angiogenic blood vessels from the host invade the injured striatum using a scaffold of glial processes derived from NSCs, as previously described during development (Campbell and Gotz, 2002). This is in agreement with previous studies that astrocytes contribute to angiogenesis, neuronal plasticity, and functional recovery from several days up to weeks after stroke (Zhao and Rempe, 2010). Thus, we may consider that grafted NSCs and their progeny could be involved in the process of angiogenesis.

### Effect of NSCs transplantation on the cellular environment after an ischemic insult

Beneficial effect of NSCs transplantation in terms of neuroprotection and functional recovery might be related to the regulation of nNOS production through functional relationships with nNOS neurons. We have found that the untransplanted homolateral striatum to ET-1 injection exhibited nNOS-positive cells showing signs of degeneration and also a higher staining intensity for nNOS than the contralateral side. In striking contrast, nNOS immunostaining in the NSCs transplanted striatum was lower and the nNOS-positive neuronal arborization was partially preserved. These results are consistent with previous results in our laboratory (Serrano et al., 2002; Alonso et al., 2002a; Encinas et al., 2004; Martinez-Murillo et al., 2007). NO produced by nNOS or the inducible isoform of NO synthase is detrimental in acute ischemic stroke (Willmot et al., 2005). NO and its derivative peroxynitrite (ONOO-), which is a potent oxidant and cytotoxic agent, inhibit mitochondrial respiration (Brown, 1999, 2003). Striatal nNOS-positive cells constitute a relatively small subpopulation of interneurons which also contain gamma aminobutyric acid (GABA), neuropeptide Y, and somatostatin (Martinez-Rodriguez et al., 1984; Vincent and Kimura, 1992; Figueredo-Cardenas et al., 1996; Sakuma et al., 2008). They appear to be relatively resistant to ischemia (Sakuma et al., 2008) and hyperexcitation to which they respond with increased NO production (Alonso et al., 2002b). Moreover knockout mice deficient in nNOS, develop stroke at a lower rate than wild type (Huang et al., 1996).

### Behavioral manifestation

Following intranigral ET-1 administration, the assessment of spontaneous motor behavior while walking and elevated tail activity were indicative of parkinsonism; specifically rotation and axial dystonia, contralateral rotations and stereotyped abnormal movements of the forelimbs. These findings are similar to parkinsonian signs described in experimental PD (Schwarting and Huston, 1996). In our model, spontaneous motor alterations decreased progressively towards the third day post-injury, despite of progressive DA degeneration as indicated by a loss of TH-positive fibres, which produce motor disfunction. The main pathological feature of PD is the loss of neurons in the SN pars compacta which leads to dopamine deficiency in the striatum and is responsible for the major symptoms of the disease. The location of DA neurons in the striatum following ET-1 injection in the SN suggests that such cells might be responsible, at least in part, for the facilitation of motor recovery. Animals with NSCs transplant exhibited improvement in a postural support task performed at 14 and 28 days after treatment when compared to the control group, which suggests the involvement of neural repair processes rather than compensatory changes in the unaffected regions of the injured striatum.

### Concluding remarks

Using a lacunar stroke experimental model induced by SN administration of ET-1 we provide morphological evidence to support the therapeutic potential of cell replacement therapy, which may corroborate a previous electrophysiological study (Buhnemann et al., 2006). Although the percentage survival of transplanted NSCs was not measured in this study, clear *in vivo* evidence for the presence of surviving NSCs which readily expressed neural and glial markers is shown. Together with the morphological integration of GFP-positive NSCs within the host tissue, the effect on angiogenesis with blood vessels adjacent to GFP-like astrocytes is remarkable. In addition, NSCs transplantation might contribute to restoration of lost motor function and cognitive deficits specifically by: (i) integrating into morphologically defined synaptic networks within the host brain, and (ii) providing trophic support for neurogenesis within and around the damaged area (c.f. Wei et al., 2005). As the fraction of lost striatal neurons replaced by endogenously generated new neurons following stroke is normally small (Arvidsson et al., 2002), we believe that transplanted NSCs could represent a promising therapeutic strategy for better reconstruction of the striatal neural circuit.

### Conflict of interest statement

The authors declare that the research was conducted in the absence of any commercial or financial relationships that could be construed as a potential conflict of interest.

## Abbreviations

ChAT: Choline acetyltransferase
DA: Dopaminergic
GFP: Green fluorescent protein
IHC: immunohistochemistry
NO: nitric oxide
nNOS: neuronal NO synthase
PD: Parkinson's disease
SN: Substantia nigra
TH: Tyrosine hydroxylase
VP: Vascular Parkinsonism
NSCs: Neural stem cells.
